# Hyperthermia inhibits cellular function and induces immunogenic cell death in renal cell carcinoma

**DOI:** 10.1186/s12885-023-11106-8

**Published:** 2023-10-12

**Authors:** Yin Huaqi, Dong Bingqi, Zhao Yanhui, Ma Yongkang, Zhao Shiming, Sun Zhenghui, Du Zheng, Peng Jiangshan, Yang Tiejun

**Affiliations:** 1 Department of Urology, The Affiliated Cancer Hospital of Zhengzhou University, Henan Cancer Hospital, No. 127, Dong Ming Road, Zhengzhou, 450000 China; 2 Department of Urology, Qingdao Central Hospital, No. 127, Si Liu Nan Road, Qingdao, 266042 China

**Keywords:** Renal cell carcinoma, Hyperthermia, Immunogenic cell death, Immune microenvironment

## Abstract

**Background:**

In recent years, hyperthermia has been widely applied as a novel strategy for cancer treatment due to its multiple antitumour effects. In particular, the potential influences of hyperthermia on the tumour immune microenvironment may improve the efficacy of immunotherapies. However, the effect of hyperthermia on renal cell carcinoma (RCC) has not been well characterized until now.

**Methods:**

In the present study, we primarily evaluated the effects of hyperthermia on cellular function via cellular proliferation, migration, invasion and apoptosis assays. In addition, the influence of hyperthermia on the immunogenicity of RCC cells was analysed using flow cytometry analysis, enzyme-linked immunosorbent assays, and immunofluorescent (IF) staining.

**Results:**

Our results demonstrate that hyperthermia significantly inhibits RCC cell proliferation, migration, and invasion and promotes cell apoptosis. In addition, we verified that hyperthermia improves the immunogenicity of RCC cells by inducing immunogenic cell death.

**Conclusion:**

Our findings suggest that hyperthermia is a promising therapeutic strategy for RCC.

## Introduction

Renal cell carcinoma (RCC) is a malignant tumour originating in the proximal tubules and accounts for approximately 90% of all kidney malignancies [1]. Approximately two-thirds of patients present with metastatic disease at initial diagnosis or after surgery [2]. Although the emergence of tyrosine kinase receptor inhibitors (TKIs), such as sunitinib and cabozantinib, has largely improved the prognosis of RCC patients, the inevitable development of resistance to these inhibitors has made it urgent to explore novel treatment strategies for RCC [3, 4].

In recent years, immunotherapy has emerged as a promising therapeutic strategy and has been widely used in the treatment of various metastatic or advanced malignancies. As a first- or second-line treatment for RCC, immune checkpoint inhibitors (ICIs), alone or in combination with TKIs, provide a survival benefit and induce a robust, continuous, and specific immune response, but unfortunately have a low objective response rate (ORR) of 20–30% [5, 6]. Therefore, improving the ORR of RCC patients to immunotherapy is vital at present. A precise understanding of the tumour immune microenvironment and the underlying mechanisms of the immune response in RCC is crucial in solving this problem.

Many randomized trials have tried to improve the ORR by combining immunotherapy with TKIs or radiotherapy, however, an increased incidence of side effects was often observed in patients who received combined therapies [7]. Recently, hyperthermia has received increasing attention for its effectiveness in fighting tumours with minimal damage to normal tissues. Hyperthermia is thought to target tumours via multiple possible mechanisms. First, hyperthermia could damage tumour cells directly by inducing the denaturation of membrane proteins and DNA synthetase. For patients with pancreatic cancer, pancreaticoduodenectomy combined with intraperitoneal hyperthermic perfusion was demonstrated to be superior to pancreaticoduodenectomy alone in overall survival and 2-year survival rates [8]. A phse III clinical trial verified that the median overall survival time of patients with ovarian cancer receiving postoperative hyperthermic intraperitoneal chemotherapy was 69.5 months which was longer than that in patients receiving normothermic intraperitoneal chemotherapy (61.3 months, p < 0.05) [9]. Second, hyperthermia could reduce the physical barrier effect of tumours by alleviating their hardness and softening their extracellular matrix, thus contributing to improved drug penetration and immune cell infiltration [10]. Third, hyperthermia could enhance the chemosensitivity and radiosensitivity of tumour cells. Recent evidence revealed that the 2- or 5-year recurrence/progression-free rate in patients with nonmuscle-invasive bladder cancer receiving intravesical hyperthermic chemotherapy was higher than that in patients receiving intravesical chemotherapy alone [11]. In addition, hyperthermia could enhance the therapeutic effectiveness of gemcitabine, 5-fluorouracil, and cisplatin on PDAC cells by decreasing the half-maximal inhibitory concentration (IC50) [12]. Finally, hyperthermia has shown promise in activating the antitumour immune response both directly and indirectly. Adkins, I et al. found that heat-treated tumour cells were more easily phagocytosed by dendritic cells (DCs) and stimulated CD4^+^ and CD8^+^ T-cell activation and proliferation [13]. Wang Z, et al. found that hyperthermia could efficiently induce antitumor immunity activation and ultimately inhibit breast cancer growth by using tumor-bearing mice model [14]. Additionally, in mouse melanoma, hyperthermia was found to promote the secretion of IL-6, IL-12 and CCL2 which contributed to the activation of the immune microenvironment [15]. Furthermore, a promising role of hyperthermia plus ICIs strategy has gained more and more attention, and many ongoing clinical trials are discovering the synergistic effect between hyperthermia and ICIs [16].

In the current study, we primarily investigated the effect of hyperthermia on RCC cellular function and cell apoptosis. Additionally, we characterized the influence of hyperthermia on the immunogenicity of RCC cells at the cellular and molecular levels simultaneously.

## Materials and methods

### Cell culture and hyperthermia treatment

The two RCC cell lines (ACHN and 769P) used in the current study were donated by Professor Xu Tao at Peking University People’s Hospital. Both cell lines were cultured in Roswell Park Memorial Institute (RPMI) 1640 medium (Gibco) with 10% foetal bovine serum (Gibco) and 1% penicillin/streptomycin (Gibco) and maintained in a cell incubator with 5% CO_2_ at 37 ℃. To subject cells to hyperthermia, culture dishes or flasks were placed in a 43 ℃ water bath for 1 h and then subsequently moved to 37 ℃ to incubate for different amounts of time as needed. Cells in the control group did not receive hyperthermia.

### Cell proliferation assay

The cell counting kit-8 assay (CCK-8, Dojinaodo) was conducted to evaluate the effect of hyperthermia on cell proliferation. Briefly, cells were first treated with hyperthermia and subsequently seeded onto 96-well plates at a density of 1.5 × 10^3^. Prior to measuring absorbance, the medium in each well was replaced with 100 µL of fresh medium supplemented with 10 µL of CCK-8 solution. The cells were incubated for 1.5 h in this solution before absorbance was measured. The absorbance at 450 nm was measured at five different time points, including 0, 24, 48, 72 and 96 h.

### Cell migration and invasion assays

Transwell invasion assays with or without Matrigel were used to assess the effect of hyperthermia on cell migration and invasion. For these assays, a total of 4 × 10^4^ hyperthermia-treated or untreated cells were plated per well. Established procedures for transwell invasion assays were followed and cell calculations were performed as previously described [2].

### Flow cytometry analysis

Flow cytometry (FACSCalibur Flow Cytometer, BD) was performed to evaluate the influence of hyperthermia on cell death and the expression of damage-associated molecular patterns (DAMPs). Before cell death was analysed, hyperthermia-treated or untreated cells were incubated at 37 ℃ for 24 h. Cell apoptosis was detected using an Annexin V-FITC apoptosis assay kit according to the manufacturer’s instructions. For DAMP analysis, cells were collected 1 h after hyperthermia and incubated with HSP70 (mouse monoclonal antibody, Biolegend, Cat# 648,003, Calif, USA), CD47 (mouse monoclonal antibody, Biolegend, Cat# 323,124, Calif, USA) and CRT (rabbit monoclonal antibody, 1 µg/ml, Abcam, Cat# ab92516, Cambridge, MA, UK) antibodies for 30 min followed by flow cytometry. The data was analysed using FlowJo 10.0.

### Immunofluorescence staining

A total of 1.0 × 10^5^ cells were plated onto glass coverslips in 6-well plates and cultured for 24 h before hyperthermia treatment. After hyperthermia treatment, cells in the different groups were incubated for an additional hour before immunofluorescence (IF) staining was performed. IF staining was conducted according to standard procedures. The following primary antibodies were used: HSP70 (mouse monoclonal antibody, 1:200, Abcam, Cat# ab47455), CRT (rabbit monoclonal antibody, 1:200, Abcam, Cat# ab92516) and CD47 (rabbit monoclonal antibody, 1:200, Abcam, Cat# ab218810).

### Enzyme-linked immunosorbent assays

Cells **(**2.0 × 10^5^) were plated onto 6-well plates and cultured for 24 h before hyperthermia treatment. Following hyperthermia treatment, cells in the different groups were incubated for an additional 6 to 24 h, after which cell supernatants were collected. HMGB1 secretion was then detected using the Human HMGB1 ELISA Kit (Liankebio, Hangzhou, China) according to the manufacturer’s instructions.

### Statistical analyses

Statistical analyses were carried out by the SPSS 19.0 package or GraphPad Prism 5.0. Data are presented as the mean ± SEM. Two-way analysis of variance was used to analyse the differences in cell proliferation and apoptosis between the two groups. The differences in migration, invasion, and HMGB1 release between the two groups were analysed using a Student’s t test. In our analysis, P < 0.05 is considered statistically significant.

## Results

### Hyperthermia inhibited the proliferation, migration and invasion of RCC cells

**Fig. 1:**
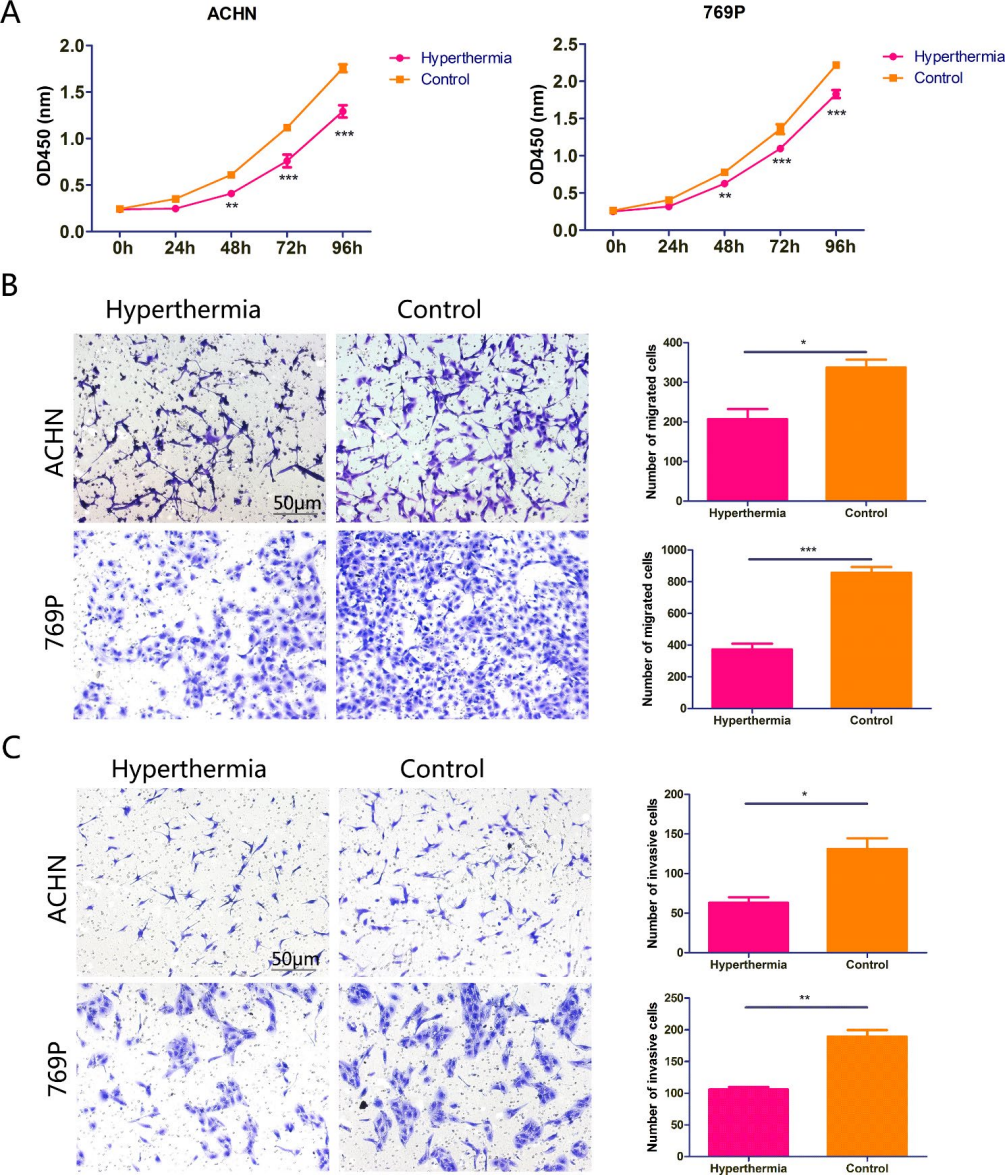
Hyperthermia significantly suppressed the proliferation, migration and invasion of RCC cells. Cellular proliferation (A), migration (B, magnification = 200×) and invasion (C, magnification = 200×) were measured using CKK-8, migration and invasion assays, respectively. *, P < 0.05; **, P < 0.01; ***, P < 0.001

To determine the influence of hyperthermia on cellular function, we performed cell proliferation, migration and invasion assays. As shown in Fig. 1A, hyperthermia significantly inhibited the proliferation of ACHN and 769P cells. Meanwhile, hyperthermia-treated cells exhibited decreased migratory and invasive abilities compared to control cells (Fig. 1B C). Taken together, these data demonstrate that hyperthermia is capable of limiting the malignancy of RCC cells.

### Hyperthermia induced immunogenic cell death in RCC cells

**Fig. 2:**
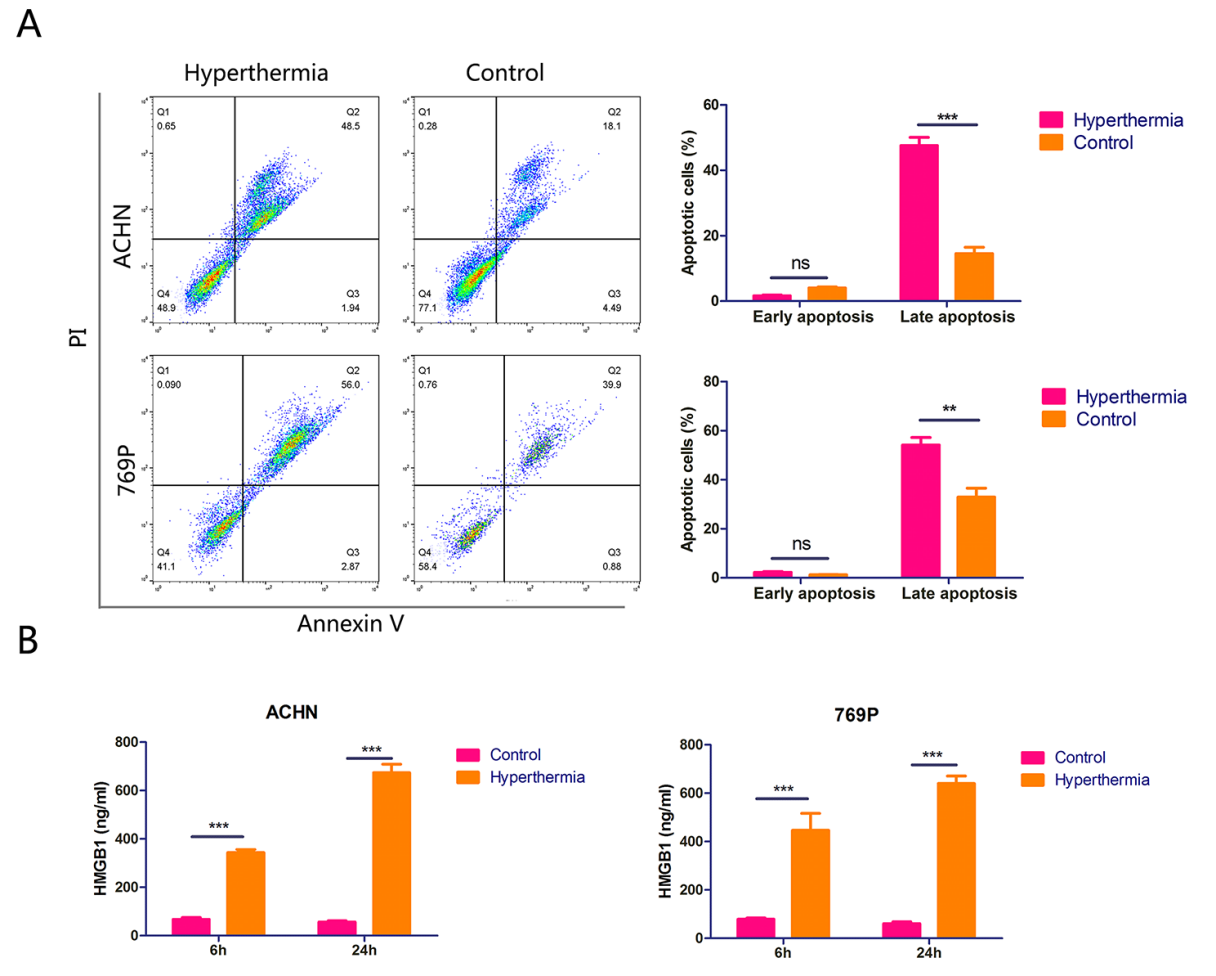
Hyperthermia treatment induced RCC cellular apoptosis and HMGB1 secretion. A, Cell apoptosis influenced by hyperthermia was assessed by flow cytometry. B, HMGB1 secretion was measured using ELISA 6 and 24 h after hyperthermia. *, P < 0.05; **, P < 0.01; ***, P < 0.001

**Fig. 3:**
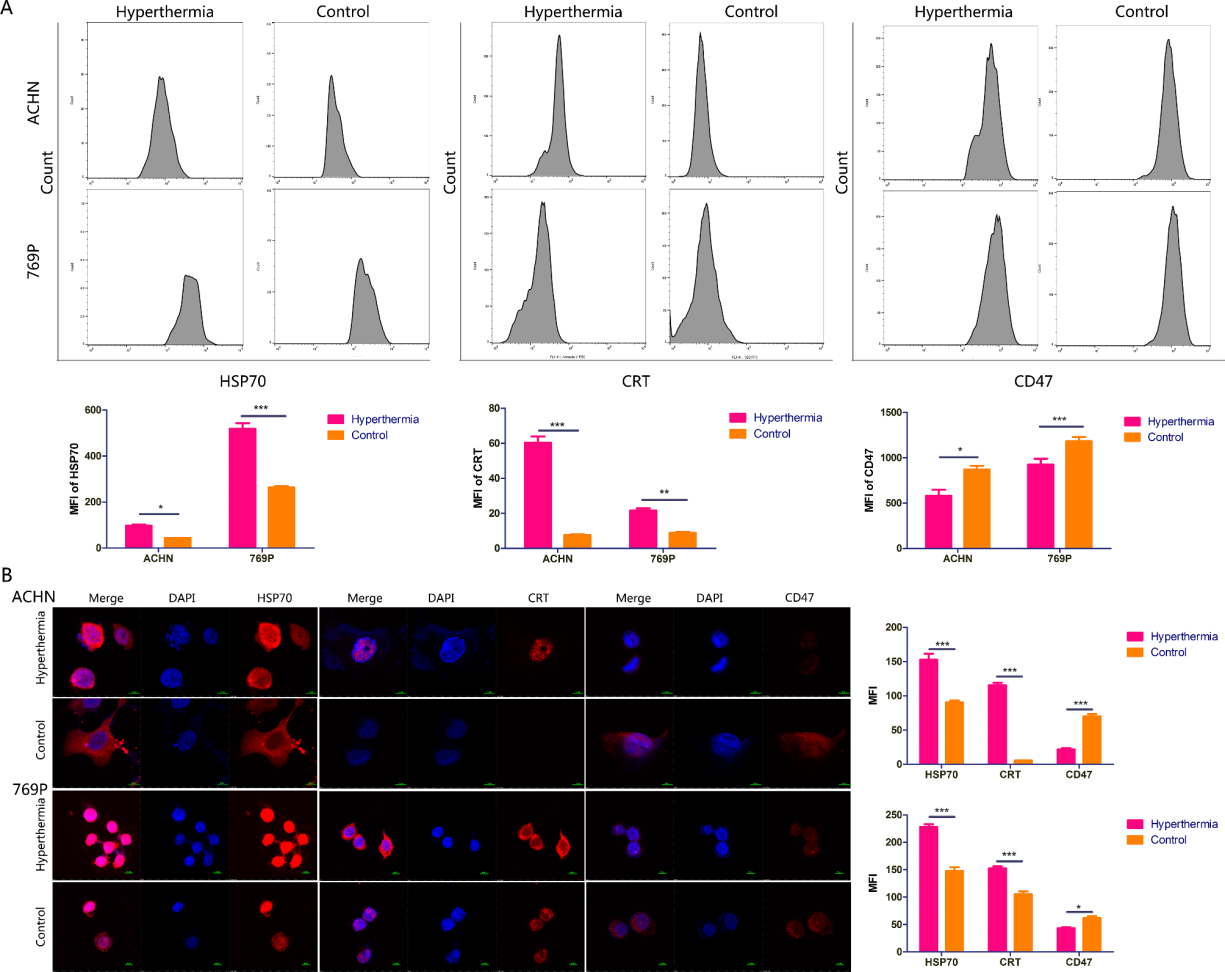
Further confirmation of the influences of hyperthermia on DAMPs. A, Flow cytometry analysis of HSP70, CRT and CD47 on the cell surface. B, IF staining of HSP70, CRT and CD47 on the cell surface (magnification = 400×). *, P < 0.05; **, P < 0.01; ***, P < 0.001

To determine whether hyperthermia treatment induced RCC cell apoptosis, we conducted flow cytometry assays. Our data revealed that the proportion of apoptotic cells in the hyperthermia group was considerably higher than that in the control group (Fig. 2A), which supports the application of local thermotherapy for tumours. We next wondered whether hyperthermia enhanced the immunogenicity of RCC cells. To test this we detected the expression of DAMPs after subjecting cells to hyperthermia. ELISA analysis revealed that HMGB1 secretion was still elevated in cells even 24 h after receiving hyperthermia treatment. This suggests hyperthermia has a prolonged effect on HMGB1 release (Fig. 2B). In addition, flow cytometry analysis showed that expression of HSP70 and CRT on the cell surface was higher after one hour of hyperthermia treatment than that observed in untreated cells, while CD47 expression showed the inverse trend (Fig. 3A). In addition, the alteration of HSP70, CRT and CD47 expression on the cell surface was further confirmed by IF staining (Fig. 3B). Collectively, these data indicate hyperthermia treatment improves the immunogenicity of RCC cells by inducing immunogenic cell death, as is supported by our observations of increased cell surface translocation of HSP70 and CRT and reduced cell surface expression of CD47.

## Discussion

Despite the various treatment approaches developed in recent years, a lack of satisfactory personalized therapies will continue to persist in the treatment of renal cell carcinoma, especially advanced RCC. The low objective response rate of immunotherapy makes it urgent to explore novel therapeutic approaches to overcome the current dilemma. Hyperthermia has been used in the treatment of various cancers since the 1970s and has a good antitumour effect [17, 18]. Among the mechanisms by which hyperthermia fights against tumours, the interaction between hyperthermia and the immune microenvironment is the most attractive in the era of immunotherapy. In vitro study verified that 60-70% of dendritic cells (DCs) could engulf heat-treated tumor cells in the co-culture system, while only 20% of DCs played phagocytosis on heat-untreated tumor cells, indicating that hyperthermia could enhance the presentation of tumor-antigen by DCs [19]. In addition, hyperthermia could induced maturation of DCs that subsequently secreted more TNF-α and IL-12p70, which in turn synergistically induced the activation and proliferation of CD4^+^ and CD8^+^T cells and enhanced the anti-tumor immune response [20]. Furthermore, in vivo study demonstrated that hyperthermia plus immunotherapy could enhance the infiltration of CD8^+^T cells and inhibit the PD-1/PD-L1 pathways [21]. Also, hyperthermia mediated by gold nanocages composed of anti-PDL1 and galunisertib contributed to improved synergistic immunotherapy in colorectal cancer patients [22]. These studies demonstrated the synergistic effects between hyperthermia and immunotherapy. However,whether hyperthermia can improve the efficacy and objective response rate of immunotherapy is still an open question.

In the current study, we found that the proliferation, migration and invasion of hyperthermia-treated RCC cells were significantly impaired. The foundation of hyperthermia treatment for malignancies lies in the widely held belief that while normal cells can survive for 1 h at 47 ℃, it is difficult for tumour cells to survive longer than 1 h at 43 ℃. Therefore, the physical stress from the 43 ℃ heat partly contributed to the inhibited cellular function. It has been verified that uncontrolled cell proliferation, migration and invasion served important roles in tumorigenesis and tumor progression of RCC. Thus, the inhibiting role of hyperthermia on cellular functions contributed to impair the malignance of RCC. In addition, hyperthermia could induce more apoptosis of RCC cells. These results demonstrated that application of hyperthermia helped to inhibit RCC progression and may bring benefits to the prognosis of patients. However, current study did not explore the molecular mechanisms by which hyperthermia regulated cellular functions of RCC, which will be further studied in the future.

Consequently, we wondered whether hyperthermia could affect the immunogenicity of RCC cells. Our data revealed that hyperthermia treatment induced elevated expression of HSP70 and CRT on the cell surface as well as reduced expression of CD47, which was further confirmed by IF analyses. In addition, hyperthermia promoted HMGB1 secretion. These essential alterations indicated activation of immunogenic cell death (ICD), which is characterized by various indices, including expression of DAMPs [23, 24]. In the process of ICD, CRT is transferred to the cell membrane and mainly transmits the “eat me” signal to antigen-presenting cells (APCs). HSP70 mainly acts as a “danger” signal together with secreted HMGB1, and the membrane protein CD47 mainly plays the role of a “do not eat me” signal in the immune response [25]. HSP70 is a member of the HSP family that may be upregulated when cells experience various shocks, including heat stress. We observed a translocation of HSP70 to the RCC cell surface under hyperthermic conditions, which is consistent with previous studies [26]. The translocation of HSP70 to the cell membrane may serve as a tumour-specific target for immune cells and help present antigens to antigen-presenting cells (APCs), subsequently activating the antitumour immune response via multiple pathways [27]. Additionally, membrane translocation of CRT and the release of HMGB1 are also vital steps in the process of ICD due to their association with enhanced antigen cross-presentation and APC maturation after hyperthermia [28]. Therefore, our data suggest that the apoptosis seen in RCC cells after hyperthermia treatment may be a form of immunogenic cell death that stokes the immune response and could improve the efficacy of immunotherapy by increasing RCC cellular immunogenicity and reprogramming the immune microenvironment. Previous studies have largely aimed to find predictive biomarkers that might indicate which patients would potentially benefit from immunotherapy. While important, such studies do not contribute to the larger goal of improving the general ORR. In the current study, we initially sought to explore an adjuvant strategy that would help to improve the ORR and efficacy of immunotherapy, even in patients with negative biomarkers. Although still far from our larger goal of improving ORR, our data demonstrate considerable promise for hyperthermia in the treatment of RCC.

## Data Availability

The data and materials supporting this study’s findings are available from the corresponding author upon reasonable request.
